# Early-Life High-Fat-Diet Exposure Induced Pre-Puberty Obesity-Related MASLD via Autophagy-Mediated Ferroptosis in Male C57BL/6J Mice

**DOI:** 10.3390/nu18091469

**Published:** 2026-05-05

**Authors:** Zihan Zhang, Yan Wu, Xiaoqing Wu, Yiyi Zhao, Chen Liang, Jinran Xu, Zhouqi Nie, Shuhan Liu, Tianni Lv, Ming Wu, Lingling Zhai

**Affiliations:** 1 Key Laboratory of Environmental Stress and Chronic Disease Control and Prevention, China Medical University, Ministry of Education, Shenyang 110021, China; zhangzihan1218t@163.com; 2 Department of Maternal and Child Health, School of Public Health, China Medical University, Shenyang 110021, China; 17759525040@163.com; 3 School of Public Health, China Medical University, Shenyang 110021, China; 18842114384@163.com (Y.W.); cliang2027@163.com (C.L.); 4 Second Clinical College, China Medical University, Shenyang 110021, China; zhaoyiyi5568@foxmail.com (Y.Z.); xujinran1334@163.com (J.X.); 13080817048@163.com (T.L.); 5 School of Medical Humanities, China Medical University, Shenyang 110021, China; 15840338422@163.com; 6 School of Nursing, China Medical University, Shenyang 110021, China; liushuhan@cmu.edu.cn; 7 Liaoning Provincial Center for Disease Control and Prevention, Shenyang 110172, China

**Keywords:** early-life, obesity, metabolic dysfunction-associated steatotic liver disease, autophagy, ferroptosis

## Abstract

Objectives: Exposure to high-fat diets in early life plays an important role in metabolic dysfunction-associated steatotic liver disease (MASLD); however, the mechanism remains unclear. In this study, we explore the role of autophagy and ferroptosis in pre-puberty obesity-related MASLD caused by high-fat diets in early life. Methods: Twenty-four male C57BL/6J mice were fed over a 6-week period, and were divided into three groups: control, lactation HFD, and lactation + post-weaning HFD group. AML12 cells were treated with 0.5 mM free fatty acids (palmitic acid:oleic acid = 1:2) for 24 h to establish an in vitro model. Metabolism, autophagy, and ferroptosis-related indicators were detected. Results: Compared to the control group, the body weight, droplet deposition of the liver, Fe^2+^, and MDA level increased significantly in the lactation + post-weaning HFD group. Impaired autophagy, ferroptosis, and AMPK/mTOR/ULK1 pathway protein expression were also found in the lactation + post-weaning HFD group. Additionally, BL-918 (activate autophagy) exposure in AML12 cells may recover FFA-induced ferroptosis and disorder of lipid metabolism. Conclusions: Early-life high-fat-diet exposure induced pre-puberty obesity-related MASLD, possibly via autophagy, which may be regulated by the AMPK/mTOR/ULK1 pathway and mediated by ferroptosis in male mice.

## 1. Introduction

Metabolic dysfunction-associated steatotic liver disease (MASLD), previously called NAFLD or MAFLD, is defined by excessive lipid deposition in hepatocytes. Early pathological alterations of MASLD are potentially reversible; however, some cases of MASLD can progress to metabolic dysfunction-associated steatohepatitis (MASH), which can further develop into hepatic fibrosis, cirrhosis, and even hepatocellular carcinoma [1]. Rodent models are mainly used in preclinical MASLD research. Some research has established an MASLD rodent model by exposing animals to high-fat diets (HFDs), which simulates the manner of exposure in humans [2,3,4]. However, the pathogenesis of MASLD caused by HFD-induced obesity has not been clearly explained. Thus, we try to examine the pathogenesis of MASLD using a mouse model, as this cannot be achieved among humans. Although adult MASLD mouse models have been established in some studies [1], a pre-puberty (equivalent to the pediatric period in humans) MASLD mouse model has not yet been well established. Male mouse models are usually used for MASLD more often than female ones because males are strongly associated with MASLD, and certain fat distribution patterns (more visceral fat) and high androgen levels, as well as increasing lipid accumulation in the liver, are found in males [5,6,7]. Thus, our study tries to discover the possible mechanisms of MASLD through a pre-puberty male mouse MASLD model.

As studies have reported, pathological damage to the liver caused by HFD may be due to the following pathways: HFD directly increases the delivery of free fatty acids (FFAs) to the liver, where the rate of synthesis exceeds that of decomposition, leading to the excessive accumulation of triglycerides (TG) in hepatocytes; long-term energy excess caused by HFD can lead to insulin resistance and mitochondrial dysfunction, then disrupt the dynamic balance between lipid oxidation and synthesis in the liver [8,9]; HFD also promotes the inflammatory and fibrotic processes by regulating intracellular homeostasis mechanisms (such as ferroptosis or autophagy); however, the underlying molecular mechanisms remain unclear [10].

Ferroptosis is a form of iron-dependent, regulated cell death and is triggered by lipid peroxidation. It plays a crucial role in the transition from MASLD to MASH. Glutathione peroxidase 4 (GPX4) is a key regulator of ferroptosis, and its downregulated expression is one of the characteristics of ferroptosis [11,12]. GPX4 removes phospholipid hydroperoxides through reduced glutathione and maintains membrane stability, while HFD inhibits the expression of GPX4 by downregulating nuclear factor E2-related factor 2 (Nrf2) signaling, enhancing the susceptibility to ferroptosis of hepatocytes [12]. Nrf2 is a transcription factor for antioxidant stress, and its stability is regulated by Kelch-like ECH-associated protein 1 (Keap1).

Zhao et al. found that autophagy is also closely associated with the development and progression of MASLD [13]. P62 serves as a selective autophagy receptor and recruits and transports intracellular substances through the autophagosome–lysosome pathway for clearance. When autophagy increases, P62 decreases, and the expression level of P62 shows a negative correlation with autophagic activity [14]. Microtubule-associated protein light chain 3 II (LC3II) is a marker for autophagy formation, and the LC3II/I ratio is commonly used to estimate the autophagy level [15]. As a core mechanism for maintaining hepatocyte homeostasis, autophagy function was regulated by the AMPK/mTOR/ULK1 signaling axis [16,17,18,19]. Ferroptosis and autophagy are both associated with the development and progression of MASLD, but we are unsure of the exact role of autophagy on ferroptosis in regulating these criteria caused by HFD.

Additionally, the mechanism of pre-puberty MASLD caused by HFD remains unknown and may differ from adult MASLD [20]. Thus, we utilized a high-fat diet to establish a pre-puberty obesity-related male MASLD mouse model (in vivo model), and an in vitro model (AML12 cells) to confirm the following hypothesis: the development of pre-puberty obesity-related MASLD may be linked to dysregulated expression of the AMPK/mTOR/ULK1 pathway in the liver. This dysregulation impairs autophagy function, exacerbates ferroptosis, elevates hepatic TG levels, and ultimately leads to lipid metabolic disorders. In addition, the pre-puberty period may include the lactation and the post-weaning stage. It has yet to be determined whether the effects of high-fat exposure on the metabolism of the liver during various pre-puberty periods are different. Therefore, two high-fat exposure groups were designed, the lactation group and the lactation + weaning group, to reveal the impact of high fat on pre-puberty lipid metabolism at different stages.

## 2. Materials and Methods

### 2.1. Subjects and Groups for Animal Experiments

#### 2.1.1. Experimental Animals

Twenty-four pregnant C57BL/6J mice (Beijing Huafu Kang Biotechnology Co., Ltd., Beijing, China) were raised by single-cage rearing in the SPF-level laboratory animal Department of China Medical University with a 12 h alternation between light and dark. The pregnant mice were fed a normal diet during the entire period of the pregnancy. The living environment and food of the pregnant mice remained consistent. After the pregnant mice gave birth and until the end of the lactation period, we called them “dam mice”. Dam mice were fed according to Table 1. When the weaning period arrived (21st day), one male offspring mouse from each litter was randomly moved to another cage. Four offspring mice were placed in each cage. Finally, 24 male offspring mice were selected for this study. During this period, the animals were free to consume feed and water. All experimental methods and procedures were reviewed and approved by the China Medical University Animal Welfare Ethics Review (Approval number: CMU2022116), laboratory animal license number: SCXK (Jing) 20190008.

The criteria for inclusion and exclusion in the experiment were followed: After they were delivered, we examined viable and healthy male neonatal mice. One male neonatal mouse was randomly selected from each litter for inclusion in the study. Dead neonatal mice at birth were excluded. 

#### 2.1.2. Animal Feed and Group

The feed diet was divided into a high-fat diet and a normal diet. The high-fat feed was provided by Nantong Terofi Feed Co., Ltd. (Nantong, China). The energy supply ratio was 60.00% fat, 19.00% protein, and 21.00% carbohydrate, and the total energy was 5.10 kcal/g. The normal feed was provided by the Department of Animal Science of China Medical University, with an energy ratio of 24.02% fat, 12.95% protein, and 63.03% carbohydrates, and a total energy of 3.44 kcal/g.

Twenty-four male mice were randomly divided into the control group (control), the lactation high-fat-diet group (lactation HFD), and the lactation + post-weaning high-fat-diet group (lactation + post-weaning HFD), with 8 mice in each group. The sample size of eight per group met the requirements of the ARRIVE 2.0 guideline. The 3R principle was strictly followed, ensuring the statistical test efficacy while avoiding unnecessary use of animals. The feeding protocols of the three groups are shown in Table 1. All mice were fed until the 6th week (42 days), which was equivalent to the pediatric period in humans.

#### 2.1.3. Process of Animal Experiment

After 6 weeks of treatment and fasting for 12 h, the mice were anesthetized with tribromoethanol (Afodin). The mice were sacrificed after blood collection, and liver and fat tissues were quickly removed. The blood samples were centrifuged at 3500 r/min to obtain serum, and the serum was frozen at −80 °C for further analysis. Part of the liver tissues were immersed in 4% paraformaldehyde for further HE staining. Another part was cut and frozen in liquid nitrogen for Oil Red O staining. Other liver tissues were placed in Eppendorf tubes and frozen at −80 °C for the subsequent experiments.

#### 2.1.4. Determination of the MASLD Model

The MASLD model was constructed by feeding mice a high-fat diet for 6 weeks (3 weeks during the lactation period and 3 weeks after the weaning period). At 6 weeks of the experiment, the body weight, serum TC, TG, AST, and ALT levels of mice had increased in the experimental group compared to the control group, and large areas of cell red staining (Oil Red O staining could reflect the lipid droplet deposition) were found in the livers of the experimental group. The above data showed the successful establishment of the pre-puberty obesity-related MASLD mouse model [6].

### 2.2. Indicators and Methods for Animal Experiments

#### 2.2.1. Detection of Body Weight, Body Composition, Organ, and Fat Coefficients

From the age of 3 weeks, the mice were weighed daily. Lean body mass and body fat were measured using a body composition analyzer.

Body fat percentage = Body fat (g)/body weight (g) × 100%.

Lean body mass percentage = lean body mass (g)/body weight (g) × 100%.

Liver coefficient = Liver weight (g)/body weight (g) × 100.

#### 2.2.2. Determination of TG, T-CHO, ALT, and AST

TG, T-CHO, ALT, and AST levels were detected using a triglyceride (TG) test kit, total cholesterol (TCH/T-CHO) test kit, aspartate aminotransferase (AST) test kit, and alanine aminotransferase (ALT) test kit. All kits were produced by the Nanjing Jiancheng Institute of Bioengineering (Nanjing, China). All operations were in accordance with the instructions of the kits. The optical densities (OD) of TG, T-CHO, ALT, and AST were detected by Microplate readers (Epoch2, Biotek, Shoreline, WA, USA), and their concentrations were calculated through standard curve extrapolation.

#### 2.2.3. HE Staining and Oil Red O Staining

HE staining was used for histological analysis, allowing visualization of the pattern of the hepatic steatosis. The liver tissue was fixed in 4% paraformaldehyde, dehydrated with gradient alcohol, transparent with xylene, impregnated with a xylene paraffin mixture (1:1) at 40 °C for 40 min, and then embedded with paraffin. Sectioning (5 μm) was achieved with a fully automatic paraffin sectioning machine (RM2255, Leica, Wetzlar, Germany), followed by dewaxing and rehydration, staining with hematoxylin solution, differentiation with 1% hydrochloric acid alcohol for 30 s, rinsing with running water for 30 min, staining with eosin solution for 5 min, after dehydration and transparency treatment, and sealing with gum. Finally, they were observed and photographed using ScanScope (Aperio CS2, Leica, Wetzlar, Germany).

Lipid droplet deposition in liver tissue was observed by Oil Red O staining. Liver tissue cryopreserved in liquid nitrogen was frozen into OTC, and the frozen sections (10 μm) were washed with PBS three times (5–10 min each time), rinsed with 60% isopropyl alcohol for 5 min, and stained in the dark with freshly prepared Oil Red O working solution (Solabao, Beijing, China) for 15 min. Finally, it was colored with 60% isopropyl alcohol, rinsed with PBS, and re-stained with hematoxylin staining solution. Then, it underwent 1–2 s differentiation with 1% hydrochloric acid alcohol, 1 h rinsing with running water for restaining, and sealing with glycerol gelatin.

#### 2.2.4. Detection of MDA and Ferrous Ions in the Liver

The degree of lipid peroxidation in the liver is reflected by detecting malondialdehyde (MDA) levels in liver tissue using the malondialdehyde colorimetric test kit produced by Elabscience (Elabscience, Wuhan, China). The procedures were conducted as described by the kit’s instructions.

The accumulation of iron ions in the liver was evaluated by detecting the concentration of ferrous ions (Fe^2+^) in the liver tissue. A total of 0.1 g of fresh liver tissue was added to 0.9 mL of the extractant and allowed to homogenize, followed by centrifugation at 12,000× *g* for 10 min, and the supernatant was removed. After determining the dilution concentration in the pre-experiment, 150 μL of the chromogenic reagent was added to 300 μL of the standard or sample, followed by incubation at 37 °C for 10 min, and centrifugation at 12,000× *g* for 10 min. A total of 300 μL of the supernatant was removed and measured at 593 nm OD value according to the ferrous ion colorimetric test kit (Elabscience, Wuhan, China). To ensure sufficient statistical confidence while minimizing the use of tissues, 6 samples were chosen for the test.

#### 2.2.5. Detection of Related Protein Expression in Livers

Western blot (WB) was used to detect the expression levels of AMPK/mTOR/ULK1, autophagy-related (LC3II/I, p62), and ferroptosis-related (Keap1/Nrf2/GPX4) proteins in livers. For Western blot analysis, 4 samples per group were adopted, which is the minimum sample size requirement for statistical analysis.

In this experiment, 0.05 g of the liver tissue was weighed and placed in 250 μL of proportionally premixed protein lysate, and the protein supernatant was separated via low-temperature high-speed centrifugation to obtain the protein stock. This was followed by electrophoresis, membrane transfer, and hybridization. The antibody ratio concentrations were as follows: GAPDH antibody (Proteintech, Wuhan, China) and GPX4 antibody (Abmart, Shanghai, China) ratio concentrations were 1:2000; AMPK antibody (Abmart, Shanghai, China), p-AMPK antibody (Abmart, Shanghai, China), mTOR antibody (Cell Signaling Technology, Danvers, MA, USA), p-mTOR (ser2448) antibody (Cell Signaling Technology, Danvers, MA, USA), ULK1 antibody (Abmart, Shanghai, China), p-ULK1 antibody (ser757) (UpingBio, Hangzhou, China), p62 antibody (Wanlai Bio, Zhengzhou, China), Nrf2 antibody (Abmart, Shanghai, China), LC3 antibody (ABclonal, Shanghai, China), and Keap1 antibody (Wanlai Bio, Zhengzhou, China) were 1:500. Then, secondary antibody hybridization was conducted, followed by luminescence detection with mix ECL chemiluminescence (Tanon, Shanghai, China). The protein band images were collected by the automatic chemiluminescence imaging system (Tanon 5500, Shanghai, China). The gray value of the target protein band was quantitatively analyzed by ImageJ 1.54p (NIH, Bethesda, MD, USA). The relative expression levels (semi-quantitative method) were used to describe protein content, that is, the ratio of target protein to the internal reference protein (GAPDH).

### 2.3. Cell Experiments

#### 2.3.1. Experimental Cells and Group

The normal mouse liver cells (AML12 cells) used in this experiment were donated by the Department of Health Inspection, School of Public Health, China Medical University. AML12 cells were cultured with DMEM/F-12 complete medium (88% DMEM medium + 10% fetal bovine serum + 1% penicamycin bispecific antibody + 1% insulin-transferrin-selenium additive (Solabao, Beijing, China). The experimental cells were divided into the following 4 groups:

The control group (control): AML12 cells were routinely cultured in a DMEM/F12 complete medium for 24 h.

The 5 μM BL-918 group (BL-918 group): AML12 cells were cultured in a DMEM/F12 complete medium containing 5 μM ULK1 agonist BL-918 (MCE, Detroit, MI, USA) for 24 h.

The 0.5 mM FFA group (FFA group): AML12 cells were cultured for 24 h in a DMEM/F12 complete medium containing 0.5 mM free fatty acids (palmitic acid:oleic acid = 1:2, Sigma-Aldrich, St. Louis, MO, USA) to construct fatty degeneration models.

The 0.5 mM FFA + 5 μM BL-918 group (BL-918 + FFA group): AML12 cells were treated with a DMEM/F12 complete medium in combination with 0.5 mM FFA and 5 μM BL-918 for 24 h.

#### 2.3.2. The Preparation of Free Fatty Acids (FFA) and BL-918 (ULK1 Agonist) Solution

In order to simulate a high-fat exposure model in animals, FFA intervention was used to establish an in vitro model. The FFA working solution: A total of 1.0 g of fatty acid-free BSA (Solabao, Beijing, China) was added to 19 mL of ddH_2_O. It was heated in a 55 °C constant-temperature water bath for 20 min until completely dissolved, and then filtered to prepare a 5% BSA solution. A total of 8 mg of NaOH was dissolved in 2 mL of ddH_2_O to prepare a 0.1 M NaOH solution. Then, 25.64 mg of palmitic acid and 31.74 μL of oleic acid, respectively, were dissolved in 1 mL of 0.1 M NaOH solution in a water bath at 70 °C for 10 min until the solution was clear and uniform. The following solutions were prepared: 100 mM of palmitic acid intermediate and oleic acid intermediate. The intermediate solutions were then mixed with the 5% BSA solution in a volume ratio of 1:19, and placed in a 70 °C water bath for 10 min to obtain 5 mM palmitic acid and 5 mM oleic acid stock solutions. The final solution was filtered through a 0.22 μm sterile filter membrane, aliquoted into Eppendorf tubes, and cryopreserved in −80 °C ultra-low temperature cryogenic storage boxes. In the case of cell delivery, 5 mM palmitic acid stock and 5 mM oleic acid stock were diluted by 1:2, creating a 0.5 mM FFA working solution using the DMEM-F12 medium.

BL-918 (ULK1 agonist, promoted autophagy) working solution: A total of 1 mg of BL-918 powder was dissolved in 0.3749 mL of DMSO (Sigma-Aldrich, St. Louis, MO, USA), which was thoroughly mixed to obtain a 5 mM BL-918 stock, followed by filtration and storage in a −80 °C ultra-low temperature freezer. For cell administration, 5 mM BL-918 stock was diluted to a 5 μM BL-918 working solution using the DMEM-F12 medium.

### 2.4. Assay Indicators and Methods for Cell Experiments

#### 2.4.1. Determination of Cell Viability, TG, and Oil Red O Staining

CCK-8 reagent (Dingguo Changsheng, Shenyang, China) was used in determining cell viability. The TG test kit (Nanjing Jiancheng Institute of Bioengineering, Nanjing, China) was used to detect the TG levels.

Cell Oil Red O staining: After discarding the culture medium from the six-well plate, the AML12 cells were washed twice with PBS, and each well was fixed with 1 mL of 4% paraformaldehyde tissue fixative for 20 min. It was then washed twice with distilled water and then soaked for 5 min in 60% isopropyl alcohol. Staining was achieved using Oil Red O working solution for 20 min, followed by rinsing with 60% isopropyl alcohol until no excess dye was left. Then it was stained with the hematoxylin solution for 1 min, rinsed with distilled water 5 times, and 1 mL of distilled water was added to each well. Observations were obtained using an inverted microscope.

#### 2.4.2. Detection of Fe^2+^ and Reactive Oxygen Species (ROS) Levels in Cells

The cells were collected, and 0.2 mL of the buffer was added to approximately 1 × 10^6^ cells. The mixture was mixed well, and lysis occurred in an ice box for 10 min, followed by centrifugation in a low-temperature high-speed centrifuge at 15,000× *g* for 10 min. The supernatant was removed for later use. The cell ferrous ion colorimetric test kit was used for Fe^2+^ concentration determination (Elabscience, Wuhan, China, E-BC-K881-M).

Assessing intracellular oxidative stress levels was achieved by detecting the relative fluorescence intensities of reactive oxygen species (ROS). Inoculated AML12 cells in 6-well plates and administered for 24 h when the density reached 60–70%. The culture medium was discarded, and a 1:1000 diluted DCFH-DA probe was added, followed by incubation in a carbon dioxide cell incubator at 37 °C for 20 min in the dark; the samples were washed three times with serum-free culture medium, and 1 mL of PBS was added to each well. Photographs were obtained using a fluorescence microscope.

#### 2.4.3. Detection of Related Protein Expression in AML12 Cells

Western blot was used to detect the expression levels of AMPK/mTOR/ULK1, autophagy-related (LC3II/I, p62), and ferroptosis-related (Keap1/Nrf2/GPX4) proteins in AML12 cells. The methods were the same as described in Section 2.2.5.

### 2.5. Statistical Analysis

All statistical analyses in this study were performed using SPSS software (version 25.0) (IBM SPSS, Inc., Chicago, IL, USA). Data were expressed as means ± SD. Independent samples t-tests were used for comparisons between two groups, and one-way analysis of variance was used for comparisons among multiple groups. Post hoc multiple comparisons were conducted using the LSD method. The test level α = 0.05.

## 3. Results

### 3.1. The Effect of High-Fat Diet on Body Weight, Liver Pathological Changes and Liver Function in Male Mice

**Figure 1:**
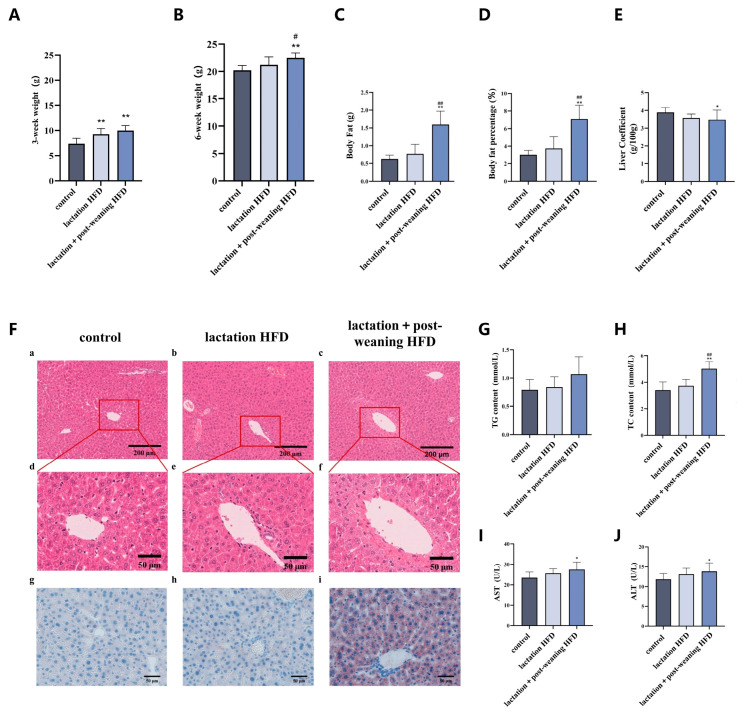
The effect of HFD on body weight, liver pathological changes and liver function in mice. (**A**): The comparison of body weight at 3 weeks (*n* = 8). (**B**–**E**): The comparison of body weight, body fat, body fat percentage and liver coefficient at 6 weeks (*n* = 8). (**F**): The pathological changes in liver tissue, (**a**–**c**): HE staining, 100×, (**d**–**f**): HE staining, 400×, (**g**–**i**): Oil Red O staining, 400×. (**G**–**J**): The comparison of serum TG, TC, AST and ALT levels (*n* = 8). Statistical analysis was conducted by one-way ANOVA followed by LSD post hoc comparison. * *p* < 0.05, ** *p* < 0.01 vs. control group; ^#^ *p* < 0.05, ^##^ *p* < 0.01 vs. lactation HFD group.

In 3 weeks, compared to the control group, the body weight of mice in the lactation high-fat-diet (lactation HFD) group and lactation + post-weaning high-fat-diet (lactation + post-weaning HFD) group significantly increased (*p* < 0.01) (Figure 1A).

In 6 weeks, compared to the control group, the body weight of mice in the lactation + post-weaning HFD group increased (*p* < 0.05) (Figure 1B), and the body fat and body fat percentage of the lactation + post-weaning HFD group were higher than those in the control group and lactation HFD group (*p* < 0.05) (Figure 1C,D). However, the liver coefficient of mice in the lactation + post-weaning HFD group was lower than that of the control group (*p* < 0.05) (Figure 1E).

Liver HE staining showed mild hepatic lobule structure disorder, scattered fat vacuoles were visible, and grade I hepatocyte swelling appeared in local areas in lactation HFD mice. And hepatocyte swelling, more disordered and congested hepatic lobule and hepatic cord structures, tortuous and stenotic hepatic sinusoids, and increased volume and number of fat vacuoles within hepatocytes were found in lactation + post-weaning HFD mice. Liver Oil Red O staining showed localized red lipid droplets in the lactation HFD group, while larger areas of red lipid droplets were found in the lactation + post-weaning HFD group (Figure 1F).

Compared with the control group, serum AST, ALT, TC and TG levels increased in lactation + post-weaning HFD mice (*p* < 0.05). All results indicated the successful establishment of the MASLD model induced by a high-fat diet in early-life male mice (Figure 1G–J).

### 3.2. The Effect of High-Fat Diet on Fe^2+^ and Ferroptosis-Related Protein in Male Mice

**Figure 2:**
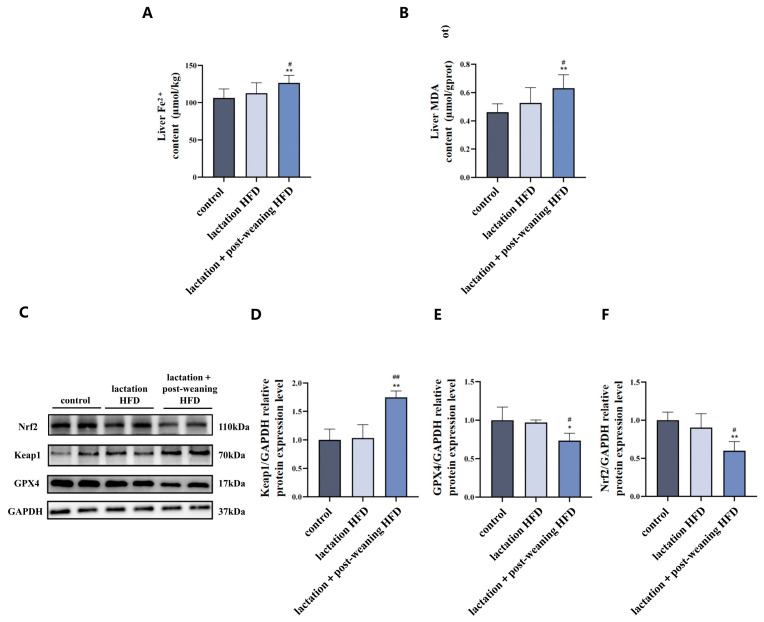
The effect of HFD on Fe^2+^, MDA and ferroptosis-related protein expression in mice. (**A**,**B**): The changes in Fe^2+^ and MDA levels (*n* = 6). (**C**–**F**): Representative Western blot band and bar graphs were used to show the results of the measurement of ferroptosis-related proteins (Nrf2, Keap1, GPX4, separately), *n* = 4. Statistical analysis was conducted by one-way ANOVA followed by LSD post hoc comparisons. * *p* < 0.05, ** *p* < 0.01 vs. control group; ^#^ *p* < 0.05, ^##^ *p* < 0.01 vs. lactation HFD group.

Iron metabolism indicator (Fe^2+^), MDA and ferroptosis-related proteins (Nrf2, Keap1 and GPX4) were detected in the livers of the three experimental groups. As compared to both the control and the lactation HFD group, the liver Fe^2+^ and MDA levels in the lactation + post-weaning HFD mice significantly increased (*p* < 0.05) (Figure 2A,B).

As shown in Figure 2C–F, in comparison with both the control and the lactation HFD group, Nrf2 and GPX4 protein expression in the lactation + post-weaning HFD group decreased, and Keap1 expression increased (*p* < 0.05).

### 3.3. The Effect of High-Fat Diet on Hepatic Autophagy in Male Mice

The autophagy substrate p62 and autophagy marker proteins LC3II and LC3I were detected while evaluating the hepatic autophagy of the livers of male mice.

**Figure 3:**
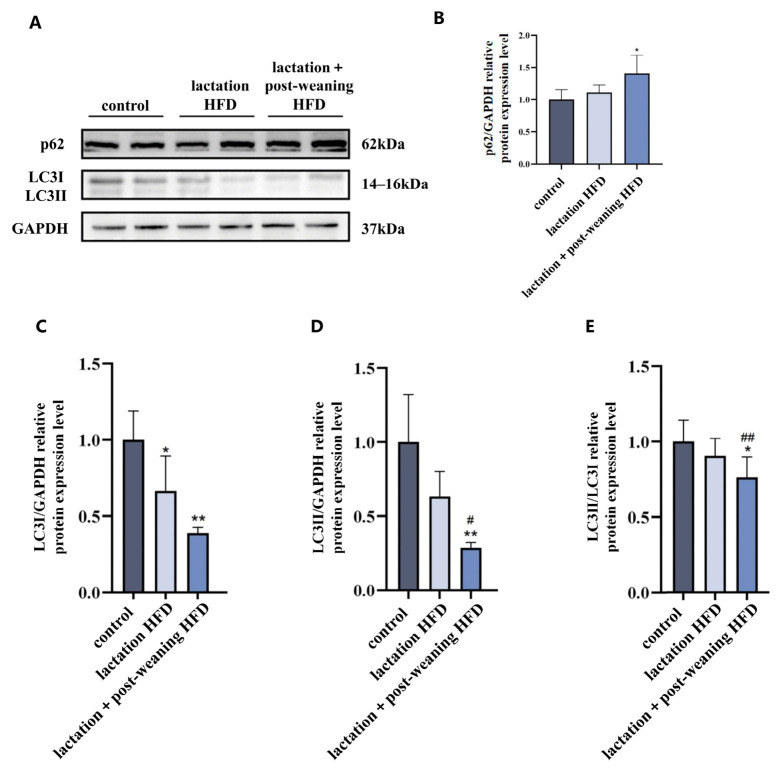
The effect of HFD on hepatic autophagy in mice. (**A**–**E**): Representative Western blot band and bar graphs were used to show the results of the measurement of autophagy-related proteins (p62, LC3I, LC3II, separately), *n* = 4. Statistical analysis was conducted by one-way ANOVA followed by LSD post hoc comparisons. * *p* < 0.05, ** *p* < 0.01 vs. control group; ^#^ *p* < 0.05, ^##^ *p* < 0.01 vs. lactation HFD group.

Compared to the control group, p62 was increased in the lactation + post-weaning HFD group, while LC3I levels decreased (*p* < 0.05). Compared to the control group, LC3II expression and its ratio to LC3I in the lactation + post-weaning HFD group decreased (*p* < 0.05). Compared to the lactation HFD group, the LC3II expression and its ratio to LC3I in the lactation + post-weaning HFD group significantly decreased (*p* < 0.01). The above results suggest that, during the lactation and post-weaning periods, continuous high-fat-diet exposure could inhibit liver autophagy, see Figure 3A–E.

### 3.4. The Effect of High-Fat Diet on Liver AMPK/mTOR/ULK1 in Male Mice

**Figure 4:**
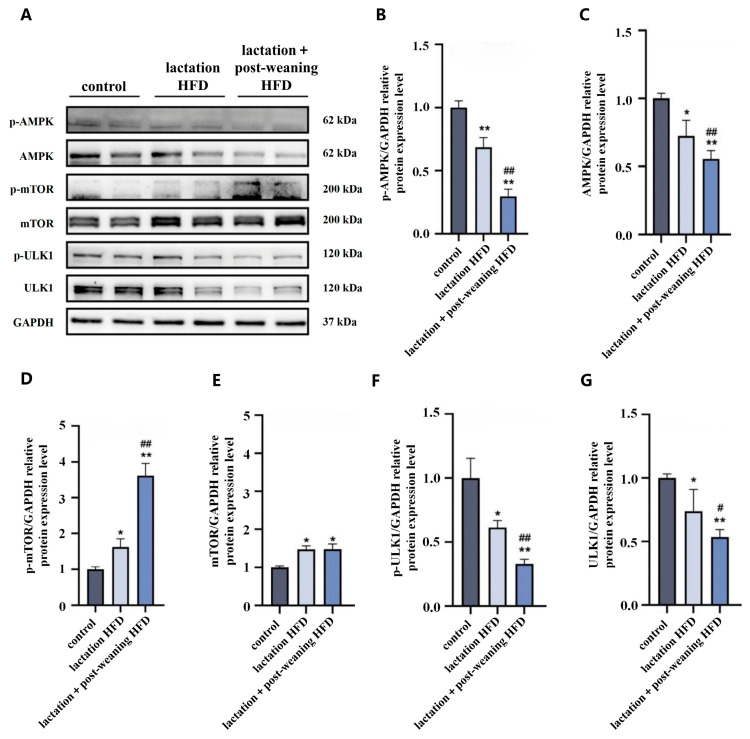
The effect of HFD on liver AMPK/mTOR/ULK1 in mice. (**A**–**G**): Representative Western blots band and bar graphs were used to show the results of the measurement of proteins of p-AMPK, AMPK, p-mTOR, mTOR and p-ULK1, ULK1, *n* = 4. Statistical analysis was conducted by one-way ANOVA followed by LSD post hoc comparison. * *p* < 0.05, ** *p* < 0.01 vs. control group; ^#^ *p* < 0.05, ^##^ *p* < 0.01 vs. lactation HFD group.

The autophagy-regulating AMPK/mTOR/ULK1 proteins were detected in the three groups. Compared to the control group, p-AMPK protein expression decreased significantly in the lactation HFD and the lactation + post-weaning HFD groups (*p* < 0.01). Compared to the lactation HFD group, p-AMPK significantly decreased in the lactation + post-weaning HFD group (*p* < 0.01) (Figure 4A–C).

Compared to the control group, p-mTOR protein expression levels increased in the lactation HFD and the lactation + post-weaning HFD groups (*p* < 0.05) (Figure 4A,D,E).

ULK1 was a key kinase of autophagy initiation. Compared to the control group, p-ULK1 protein expression levels significantly decreased in both experimental groups (*p* < 0.01). Furthermore, compared to the lactation HFD group, p-ULK1 levels significantly decreased in the lactation + post-weaning HFD group (*p* < 0.01) (Figure 4A,F,G).

### 3.5. The Changes in ROS Levels and Fe^2+^ Concentration in AML12 Cells Treated with FFA

**Figure 5:**
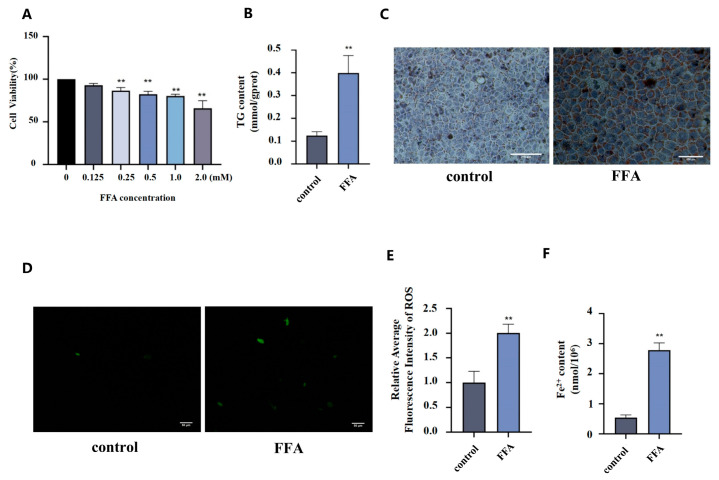
The changes in ROS levels and Fe^2+^ concentration in AML12 cells treated with FFA. (**A**): The effect of FFA on AML12 cell viability (*n* = 3). (**B**): The change in TG levels (*n* = 3). (**C**): The morphological changes in AML12 cells (Oil Red O staining, 200×). (**D**,**E**): Mean fluorescence intensity of ROS in the control and FFA groups, *n* = 3, 200×. (**F**): The change in Fe^2+^ level, *n* = 3. Statistical analysis was conducted by independent samples *t*-tests for two-group comparison, and one-way ANOVA followed by LSD post hoc comparison. ** *p* < 0.01 vs. control group.

As shown in Figure 5A, CCK-8 assay results showed that, compared to the control group, cell viability in 0.25 mM, 0.5 mM, 1.0 mM, and 2.0 mM FFA groups significantly decreased (*p* < 0.01). Based on a comprehensive consideration of model effectiveness and maintenance of sufficient cell viability, 0.5 mM FFA was selected as the induction concentration for subsequent experiments. AML12 cells were treated with 0.5 mM FFA for 24 h, and triglyceride (TG) level was measured. Compared to the control group, the TG level in FFA group significantly increased (*p* < 0.01) (Figure 5B). Oil Red O staining showed that, compared to the control group, a large number of orange-red lipid droplet deposits were observed in FFA group (Figure 5C). These morphological observations were consistent with biochemical indicators, indicating that treatment with 0.5 mM FFA for 24 h successfully induced steatosis in AML12 cells. The hepatic steatosis model was established and was used for subsequent cell experiments.

Compared to the control group, the fluorescence intensity was markedly enhanced and the number of fluorescence-positive cells significantly increased in FFA group (Figure 5D). Quantitative analysis of ROS fluorescence levels showed that, compared to the control group, the average fluorescence intensity of ROS significantly increased in FFA group (*p* < 0.01) (Figure 5E). Compared to the control group, intracellular Fe^2+^ in FFA group significantly increased (*p* < 0.01) (Figure 5F).

### 3.6. The Changes in Ferroptosis-Related Protein, Autophagy Indicators in AML12 Cells

**Figure 6:**
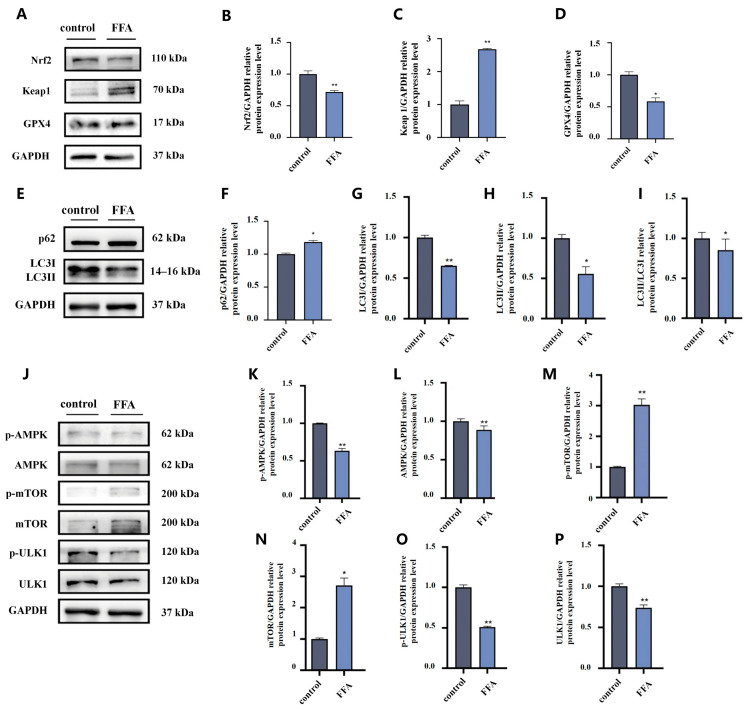
The changes in ferroptosis-related proteins and autophagy indicators in AML12 cells. (**A**–**D**): Representative Western blot band and bar graphs were used to show the results of the measurement of ferroptosis-related proteins (Nrf2, Keap1, GPX4, separately). (**E**–**I**): Representative Western blot band and bar graphs were used to show the results of the measurement of autophagy-related proteins (p62, LC3I, LC3II, separately). (**J**–**P**): Representative Western blot band and bar graphs were used to show the results of the measurement of p-AMPK, AMPK, p-mTOR, mTOR and p-ULK1, ULK1, *n* = 3. Statistical analysis was conducted by independent samples *t*-test for two-group comparisons. * *p* < 0.05, ** *p* < 0.01 vs. control group.

As shown in Figure 6A–D, compared to the control group, Nrf2 and GPX4 protein expression in the FFA group significantly decreased, whereas Keap1 protein expression increased (*p* < 0.05) (Figure 6A–D).

Compared to the control group, the autophagy substrate p62 protein expression level of the FFA group significantly increased (*p* < 0.05), while key autophagosome formation LC3I and LC3II protein expression and their ratio LC3II/LC3I decreased (*p* < 0.05). This indicates that autophagy was inhibited by the FFA intervention (Figure 6E–I).

Compared to the control group, p-AMPK level significantly decreased; however, the level of p-mTOR increased in the FFA group (*p* < 0.05). The key autophagy-initiating kinase p-ULK1 expression significantly decreased (*p* < 0.01) (Figure 6J–P).

### 3.7. The Effect of ULK1 Activator BL-918 on AML12 Cells

**Figure 7:**
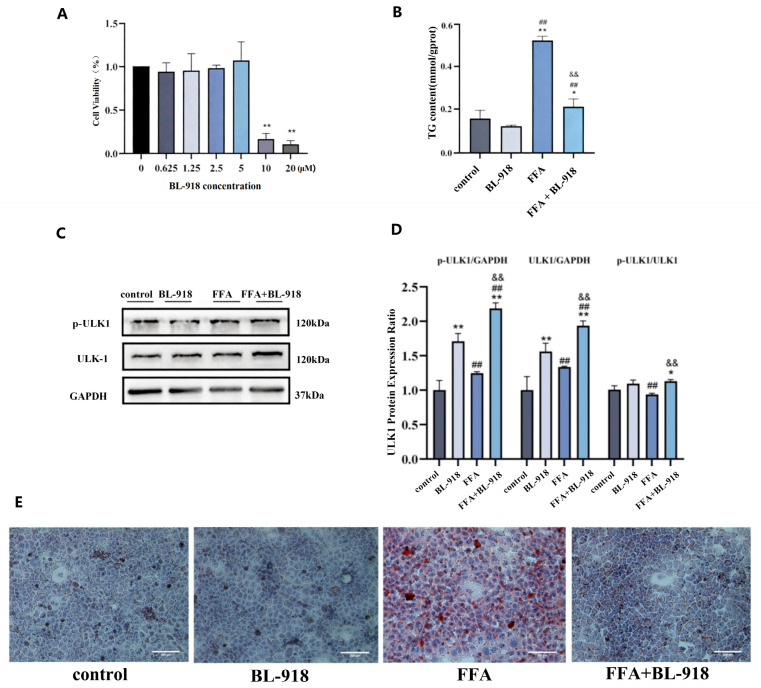
The effect of ULK1 activator BL-918 on AML12 cells. (**A**): The effect of BL-918 on AML12 cells viability, *n* = 3. (**B**): The change in TG level, *n* = 3. (**C**,**D**): Representative Western blot band and bar graphs were used to show the results of the measurement of p-ULK1 and ULK1 proteins, *n* = 3. (**E**): The lipid droplet accumulation changes in AML12 cells, Oil Red O staining, 200×. Statistical analysis was conducted by one-way ANOVA followed by LSD post hoc comparison. * *p* < 0.05, ** *p* < 0.01 vs. control group; ^##^ *p* < 0.01 vs. BL-918 group; ^&&^ *p* < 0.01 vs. FFA group.

The ULK1 activator BL-918 was used to activate autophagy in AML12 cells. As shown in Figure 7A, CCK-8 assay results showed that there were no significant changes in cell viability in the 0.625–5 μM BL-918 groups compared to the 0 μM BL-918 group. However, cell viability decreased in the 10, 20 μM BL-918 group (*p* < 0.05). A total of 5 μM BL-918 was selected for subsequent experiments.

Compared to the control group, TG levels were significantly increased in both the FFA group and the FFA + BL-918 group (*p* < 0.05). However, compared to the FFA group, TG levels in the FFA + BL-918 group significantly decreased (*p* < 0.01) (Figure 7B).

Compared to the control group, p-ULK1 protein levels significantly increased in the BL-918 group (*p* < 0.01), indicating its effective activation of ULK1. Compared to the control group, p-ULK1 expression was inhibited in the FFA group (*p* < 0.01), while p-ULK1 increased in the FFA + BL-918 group (*p* < 0.01) (Figure 7C,D).

Compared to the control group, there were no obvious lipid droplets in the BL-918 group, whereas the FFA group exhibited extensive orange-red lipid droplet accumulation. Compared to the FFA group, the lipid droplet accumulation was significantly decreased, with only localized deposition observed in the FFA + BL-918 group, indicating that BL-918 may alleviate FFA-induced steatosis. (Figure 7E).

### 3.8. The Effect of BL-918 on Autophagy, Ferroptosis in AML12 Cells

**Figure 8:**
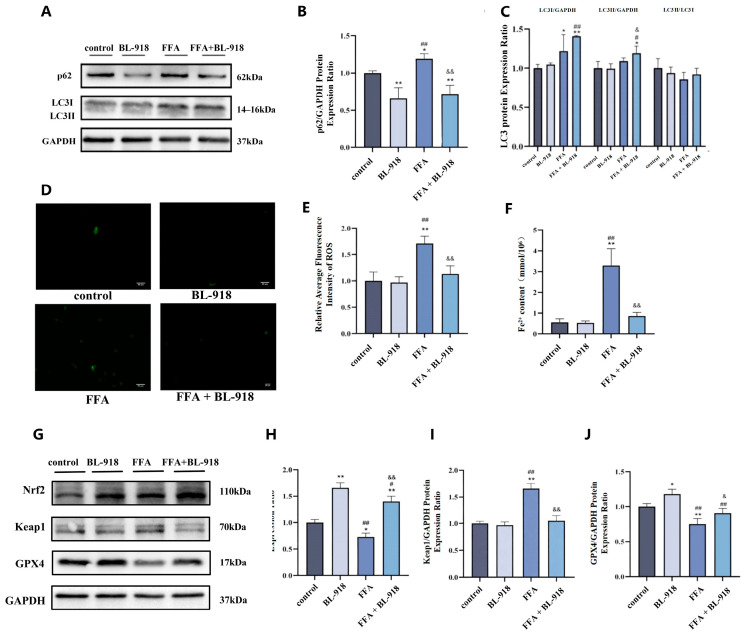
The effect of BL-918 on autophagy and ferroptosis-related proteins in AML12 cells. (**A**–**C**): Representative Western blot band and bar graphs were used to show the results of the measurement of autophagy-related proteins (p62, LC3I, LC3II, separately), *n* = 3. (**D**,**E**): Mean fluorescence intensity of ROS in four groups, *n* = 3, 200×. (**F**): The change in Fe^2+^ level, *n* = 3. (**G**–**J**): Representative Western blot band and bar graphs were used to show the results of the measurement of ferroptosis-related proteins (Nrf2, Keap1, GPX4, separately), *n* = 3. Statistical analysis was conducted by one-way ANOVA followed by LSD post hoc comparisons. * *p* < 0.05, ** *p* < 0.01 vs. control group; ^#^ *p* < 0.05, ^##^ *p* < 0.01 vs. BL-918 group; ^&^ *p* < 0.05, ^&&^ *p* < 0.01 vs. FFA group.

Compared to the control group, autophagy substrate p62 protein levels significantly increased in the FFA group (*p* < 0.05). Compared to the FFA group, p62 levels in the FFA + BL-918 group were significantly decreased (*p* < 0.01). Additionally, compared to the control group, LC3I protein levels significantly increased in the FFA + BL-918 group (*p* < 0.01), and the LC3II level of the FFA + BL-918 group significantly increased compared to that of the FFA group (*p* < 0.05) (Figure 8A–C).

Compared to the control group, the ROS fluorescence intensity in the FFA group was strong, with a high number of positive cells (*p* < 0.01). Compared to the FFA group, the ROS fluorescence intensity significantly decreased in the FFA + BL-918 group (*p* < 0.01), becoming close to the level of the control group (Figure 8D,E). Compared to the control group, intracellular Fe^2+^ significantly increased in the FFA group (*p* < 0.01), and compared to the FFA group, Fe^2+^ content significantly decreased in the FFA + BL-918 group (*p* < 0.01). (Figure 8F). Compared to the control group, Nrf2 protein expression levels significantly increased in both the BL-918 and FFA + BL-918 groups (*p* < 0.01). Furthermore, compared to the FFA group, Nrf2 levels significantly increased in the FFA + BL-918 group (*p* < 0.01), and the changes in GPX4 expression followed a trend consistent with Nrf2 (*p* < 0.01). Compared to the control group, Keap1 levels significantly increased in the FFA group (*p* < 0.01). In contrast, compared to the FFA group, Keap1 levels significantly decreased in the FFA + BL-918 group (*p* < 0.01) (Figure 8G–J). In all, activating autophagy could inhibit ferroptosis.

## 4. Discussion

MASLD may evolve to hepatic steatosis, fatty hepatitis, and even develop into liver cirrhosis and hepatocellular carcinoma [10]. MASLD pathogenesis follows the theory of the “multi-hit hypothesis”, involving lipid metabolism imbalance, oxidative stress, inflammation, and other complex mechanisms. However, the mechanisms of pe-puberty MASLD have yet to be clearly explained, and may differ from adult MASLD in mice [1]. In this experiment, the early life of the MASLD C57BL/6J mouse model was established by exposing them to a high-fat diet as in a prior mouse study [7]. Meanwhile, different periods (the lactation and the post-weaning period) were designated for high-fat-diet exposure to find the key period regulating pre-puberty MASLD.

At 6 weeks, body weight, body fat, and TG level increased; larger areas of red lipid droplets were found in hepatocytes in lactation + post-weaning HFD mice. These results were consistent with MASLD model characteristics in previous studies [21,22], indicating the successful establishment of the early-life (pre-puberty) HFD-induced MASLD mouse model.

There is a lack of evidence regarding differences in lipid metabolism mechanisms upon exposure to a high-fat diet in the lactation or lactation + the post-weaning stage. Furthermore, there are relatively limited comparative studies in this area. In our experiment, MASLD was established in the lactation + the post-weaning group, not in the lactation group. Exposure of the mother to a high-fat diet during the lactation period, leading to exposure of the offspring to a high-fat diet through milk, had a weak impact on the offspring, which may be due to the metabolism of an HFD in the mother’s body [23]. We may not need to worry about the high-fat exposure of mothers during the lactating period, but more attention should be paid to the offspring’s self-feeding period. The self-feeding period after the birthing stage may be a critical period for interventions for MASLD in children.

In our experiment, the liver coefficient of lactation + post-weaning HFD mice decreased, which may be related to liver fat deposition and metabolic abnormality, indicating that early-life HFD may affect the normal metabolism and structure of the liver. Thus, the serum TC, AST, and ALT levels were detected, showing a significant increase, which was consistent with that described by Jeong et al. [6]. This indicated that early-life HFD can lead to impaired liver function and lipid metabolism disorders in mice. But the mechanism of a high-fat diet on lipid metabolism disorders was not clearly explained in the early-life stage. Autophagy and ferroptosis in the liver were mentioned to explore pre-puberty obesity-related MASLD mechanisms in this study.

In this study, we found that Fe^2+^ and MDA contents in the liver both increased, and ferroptosis-related Nrf2 and GPX4 protein expression levels decreased; however, Keap1 expression increased. Our research results were consistent with those of Chen et al. [11]. Nrf2 is a crucial transcription factor in antioxidant stress responses by binding to antioxidant response elements in antioxidant gene promoter regions and promoting antioxidant enzyme expression, which helps maintain cellular redox balance. Keap1 is a negative regulator of Nrf2, maintaining low-level Nrf2 expression by mediating its ubiquitination and degradation. When exposed to an HFD, a decrease in Nrf2 (inhibited by Keap1) can downregulate GPX4 expression, with ferrous ion accumulation and increased MDA levels, which may enhance the susceptibility of hepatocytes to ferroptosis [17,19]. The early life of HFD could induce hepatic ferroptosis.

Ferroptosis is a cell death form driven by lipid peroxidation. During this process, intracellular free Fe^2+^ levels significantly increase, and ferrous ions catalyze the conversion of hydrogen peroxide into hydroxyl radicals through the Fenton reaction, further promoting lipid peroxidation. The lipid peroxidation product MDA is induced by elevated ROS levels, which serve as a critical ferroptosis biochemical characteristic. GPX4 is a key regulatory factor of ferroptosis, with downregulated expression as one of ferroptosis’s characteristic features. It inhibits ferroptosis by scavenging phospholipid hydroperoxides through reduced glutathione to maintain cell membrane stability. However, we are unsure which one could regulate hepatic ferroptosis in those exposed to HFD in early life.

In this study, we also found that P62 expression increased, while LC3II/LC3I expression decreased. As an autophagy receptor, p62 accumulation usually indicates impaired autophagic function, and LC3II/LC3I ratio decrease suggests impaired autophagosome formation and inhibited autophagic flux at the degradation stage, which highlights that hepatic autophagic function could be inhibited by early-life HFD. Autophagy is an intracellular self-degradation process that is crucial for maintaining intracellular homeostasis. In normal contexts, autophagy removes dysfunctional intracellular components through lysosomal degradation, including damaged organelles and protein aggregates, thereby maintaining the normal function of cells. In this study, autophagic function was impaired, which may lead to excessive lipids and lipid metabolism disorders.

Autophagy is a core mechanism for maintaining hepatic cell homeostasis that can be regulated by the AMPK/mTOR/ULK1 signaling axis [24]. ULK1 is a key kinase in the autophagic process, and its activity is regulated by its cellular energy status. In normal contexts, AMPK is activated when the cell is at a low energy level and can inhibit mTOR activity. MTOR is a negative regulator of autophagy. Once mTOR activity is suppressed, ULK1 inhibition is relieved, thereby activating ULK1, initiating the autophagic process, and maintaining intracellular energy balance and metabolic homeostasis. However, intracellular energy metabolism becomes dysregulated and the activation of AMPK is inhibited upon exposure to a high-fat diet. This leads to sustained mTOR activation, which excessively suppresses ULK1, hindering autophagy initiation, blocking autophagic flux, and thus impairing the effective clearance of excess intracellular lipids and damaged organelles. p-AMPK expression decreases, p-mTOR expression increases, and p-ULK1 expression decreases. These changes are consistent with the findings of previous studies [25]. In sum, abnormal expression of AMPK/mTOR/ULK1 could decrease autophagic function.

Because it is unknown if autophagy can regulate hepatic ferroptosis, cell experiments were conducted to verify the possible mechanism. The ULK1 inhibitor BL-918 (activated autophagy) was used to verify the regulatory role of autophagy on ferroptosis. We found that BL-918 can recover HFD-induced steatosis. Meanwhile, BL-918 could reduce the intracellular Fe^2+^ level, the ROS fluorescence relative mean intensity, and Keap1 protein expression level, while increasing Nrf2 and GPX4 protein expression levels, indicating that it can alleviate HFD-induced ferroptosis. Therefore, early-life high-fat-diet exposure could induce pre-puberty obesity-related MASLD, possibly via autophagy, which may be regulated by the AMPK/mTOR/ULK1 pathway and mediated by ferroptosis in male mice.

There are some limitations and strengths in our study. Firstly, the selection of animal samples did not involve female mice; we will include female mice in our subsequent research. Secondly, in our cell rescue experiment, only the ULK1 agonist (BL-918) was used, but other possible intervention targets and drugs were not applied. Thirdly, direct assessment of autophagic fluxes was not performed in this study, and should be conducted in future studies, although the P62 and LC3II/LC3I ratio could reflect the level of autophagic flux. Fourthly, an early-life MASLD (pre-puberty period) model was established, which could improve the MASLD model from the pre-puberty to the adult period. Lastly, a potential target may have been identified (ULK1 agonist), which could be referred to as a target for pre-puberty MASLD intervention. However, human research should be executed when extrapolating these target findings from animals to humans.

## 5. Conclusions

In conclusion, MASLD can occur in male mice exposed to a high-fat diet during the lactation and weaning periods. Abnormal expression of AMPK/mTOR/ULK1 decreases autophagy function, possibly aggravates ferroptosis, and may lead to an increase in TG levels, which may be related to MASLD caused by a high-fat diet during the pre-puberty period.

## Figures and Tables

**Table 1:** The feeding protocols for the three groups of dam and male mice.

Graph Name	Period		Total Time
	In Lactation (1st–3rd w)	In Post-Weaning (4th–6th w)	
Control Group	Dam mice fed normal diet for 3 weeks	Offspring mice fed normal diet for 3 weeks	6 weeks
Lactation HFD Group	Dam mice fed HFD for 3 weeks	Offspring mice fed normal diet for 3 weeks	6 weeks
Lactation + post-weaning HFD Group	Dam mice fed HFD for 3 weeks	Offspring mice fed HFD for 3 weeks	6 weeks

## Data Availability

The original contributions presented in this study are included in the article; further inquiries can be directed to the corresponding authors.

## Abbreviations

The following abbreviations are used in this manuscript:

ALT	Alanine aminotransferase
AMPK	AMP-activated protein kinase
AST	Aspartate aminotransferase
FFA	Free fatty acids
GPX4	Glutathione peroxidase 4
HFD	High-fat diet
Keap1	Kelch-like ECH-associated protein 1
LC3	Microtubule-associated protein light chain 3
MASH	Metabolic dysfunction-associated steatohepatitis
MASLD	Metabolic dysfunction-associated steatotic liver disease
NAFLD	Non-alcoholic fatty liver disease
MDA	Malondialdehyde
mTOR	Mammalian target of rapamycin
NCDs	Non-communicable diseases
Nrf2	Nuclear factor erythroid 2-related factor 2
ROS	Reactive oxygen species
T-CHO	Total cholesterol
TG	Triglycerides
ULK1	Unc-51-like autophagy activating kinase 1
